# Neighborhood Deprivation, Antibiotic Prescribing Concordance, and 30-Day Outcomes After Emergency Department Encounters in Older Adults

**DOI:** 10.1093/ofid/ofag455

**Published:** 2026-07-23

**Authors:** Mayar Al Mohajer, David Slusky, David E Nix, Kasim Allel, Sabra Shay, Catia Nicodemo

**Affiliations:** Department of Medicine, Baylor College of Medicine, Houston, Texas, USA; Department of Continuing Education, University of Oxford, Oxford, UK; Nuffield Department of Primary Health Care and Science, University of Oxford, Oxford, UK; Department of Economics, University of Kansas, Lawrence, Kansas, USA; National Bureau of Economic Research, Cambridge, Massachusetts, USA; IZA—Institute of Labor Economics, Bonn, Germany; Department of Pharmacy Practice and Science, University of Arizona, Tucson, Arizona, USA; Nuffield Department of Primary Health Care and Science, University of Oxford, Oxford, UK; Premier Inc., Colorado Springs, Colorado, USA; Nuffield Department of Primary Health Care and Science, University of Oxford, Oxford, UK; IZA—Institute of Labor Economics, Bonn, Germany; Brunel Business School, Brunel University of London, London, UK

**Keywords:** antibiotic overuse, antimicrobial stewardship, emergency department, neighborhood deprivation, older adults

## Abstract

**Background:**

Older adults evaluated in emergency departments (EDs) frequently receive antibiotics, but the relationships among neighborhood deprivation, antibiotic decision concordance, and downstream outcomes remain incompletely defined.

**Methods:**

We conducted a retrospective cohort study of 1 365 957 ED encounters among adults aged ≥65 years at 119 CommonSpirit Health facilities across 15 US states (2015–2024). Census-tract Social Deprivation Index (SDI) was standardized for modeling. Indication-level concordance, overuse, and underuse were based on antibiotics administered during the index ED encounter and an encounter-level diagnosis-tier framework. Outcomes were length of stay (LOS), 30-day ED revisit, mortality, *Clostridioides difficile* infection (CDI), and desirability of outcome ranking (DOOR).

**Results:**

Associations between higher SDI and outcomes were statistically detectable but small in absolute magnitude. Guideline-concordant management was associated with shorter LOS, better DOOR, and lower revisit, mortality, and CDI risks; overuse was consistently associated with worse outcomes, whereas underuse showed mixed associations. Concordance mediated only a small share of SDI-associated outcome differences. Sensitivity analyses using a 4-level DOOR outcome, modified Poisson relative risks, and study-period stratification supported the principal interpretation.

**Conclusions:**

Neighborhood deprivation had small absolute associations with short-term outcomes. Indication-level concordance and avoidance of overuse were associated with more substantial outcome differences but explained little of the deprivation-associated variation.

## Teaser

Neighborhood deprivation showed small absolute associations with short-term outcomes after ED encounters among older adults. Indication-level antibiotic concordance, particularly avoidance of overuse, was associated with larger outcome differences but mediated little of the deprivation–outcome association.

Unnecessary antibiotics in acute care remain a key target for antimicrobial stewardship because of the association with antimicrobial resistance (AMR) and adverse impact on morbidity and mortality. Unnecessary antibiotics are associated with preventable harms, including *Clostridioides difficile* infection (CDI) and drug-related adverse events [1, 2]. Older adults are particularly vulnerable given multimorbidity, polypharmacy, and age-related physiologic changes that increase susceptibility to antibiotic-related toxicity and dysbiosis [3, 4].

Emergency departments (EDs) are an important setting for antibiotic initiation in older adults, where prescribing decisions are made under time pressure and with imperfect diagnostic certainty. In national ED data from 2016 to 2021, antibiotics were prescribed in 18.6% of visits, and nearly 1 in 3 prescriptions lacked guideline support; among older adults, 24.6% of antibiotic prescriptions were classified as inappropriate [5]. Improving appropriateness requires attention to 2 distinct types of misuse. Overuse exposes patients to immediate harms without clinical benefit, whereas underuse delays indicated therapy and may increase the risk of clinical deterioration when bacterial infection is present. However, stewardship metrics and much of the published literature have emphasized overuse and total prescribing volume, leaving underuse less visible despite its potential consequences [6, 7].

Broader social and health system contexts also shape prescribing behavior and downstream outcomes. The Social Deprivation Index (SDI) quantifies community disadvantage and may capture structural factors related to delayed presentation, barriers to follow-up, and differential access to diagnostics and care [8, 9]. Place-based inequities in timely access to services have been documented across conditions and settings, including spatial accessibility to COVID-19 testing and patterns of prehospital delay for time-sensitive conditions [10, 11]. Prior studies have applied neighborhood indices to describe inequities in antibiotic prescribing and access [12–14]. In our related prior analysis of this ED network, higher area deprivation was associated with lower guideline-concordant antibiotic prescribing, primarily through underuse [15]. Whether those prescribing differences translate into downstream patient-centered outcomes, and the extent to which concordant prescribing mediates any deprivation–outcome association, remain unclear.

In this analysis, we examined associations of neighborhood deprivation (SDI) and antibiotic prescribing concordance—overall and partitioned into overuse and underuse—with 30-day outcomes after ED encounters among older adults, including length of stay (LOS), 30-day ED revisit, 30-day mortality, 30-day CDI, and an ordinal composite desirability of outcome ranking (DOOR) score. We additionally used mediation analyses to estimate the proportion of the SDI–outcome association that operates through guideline concordance to clarify the degree to which stewardship may improve outcomes and advance equity.

## METHODS

### Study Design and Data Sources

We conducted a retrospective cohort study of ED encounters at 119 CommonSpirit Health facilities across 15 US states (Arkansas, Arizona, California, Georgia, Iowa, Kentucky, Minnesota, North Dakota, Nebraska, Nevada, Ohio, Oregon, Tennessee, Texas, and Washington) between January 1, 2015, and December 31, 2024. Encounter-level electronic health record (EHR) data—not administrative claims or Premier charge data—were obtained through TheraDoc (Premier, Inc., Charlotte, NC) and included demographics (age, sex, and race/ethnicity), comorbidities, vital signs, disposition, antibiotic administrations, diagnosis codes, and patient and facility addresses. Census-tract–level contextual measures were linked using the patient census tract. Social deprivation was measured using the SDI, which is derived from American Community Survey indicators and incorporates poverty, low educational attainment, nonemployment, single-parent households, renting, overcrowding, and lack of vehicle access [8, 9]. Hospital characteristics were supplemented using the American Hospital Association Annual Survey Database [16], national sources for teaching hospital identification [17], and Rural–Urban Commuting Area codes based on facility ZIP code [18]. Travel time from the patient residence to the facility was calculated using OpenStreetMap-based routing methods [19].

### *Study* Sample

We included ED encounters among adults aged ≥65 years during the study period with ICD-9 or ICD-10 diagnosis codes recorded in the source EHR. All qualifying encounters were included regardless of ED disposition; encounters admitted from the ED remained in analyses of LOS, mortality, CDI, and DOOR, whereas the ED-revisit outcome was restricted to encounters discharged from the ED. Encounters were excluded if patient residence was outside the 50 US states and the District of Columbia or if patient or facility address data were missing and therefore could not be linked to census-tract measures.

### Exposures

The source SDI score ranges from 1 to 100, with higher values indicating greater deprivation. For analytic modeling, the available working SDI index was standardized to a *z*-score so estimates represent a 1-SD increase; negative standardized values denote deprivation below the cohort mean and positive values denote deprivation above the cohort mean. For descriptive analyses, SDI was categorized into quintiles, and original-scale values are summarized in the Supplementary Materials.

Antibacterial use was defined from any antibacterial administration recorded during the index ED encounter; discharge prescriptions and antibiotics initiated after ED discharge were not captured. We evaluated indication-level guideline concordance and its nonconcordant components, overuse and underuse. Concordance was assigned using a previously published encounter-level ICD-9/ICD-10 diagnosis-tier framework informed principally by Infectious Diseases Society of America guidance available before December 2024 [5, 15, 20]. Tier 1 included diagnoses for which systemic antibiotics are generally indicated (eg, bacterial pneumonia, cystitis/urinary tract infection, sepsis, and cellulitis), Tier 2 included diagnoses for which antibiotics may be indicated depending on clinical context, and Tier 3 included diagnoses for which antibiotics are generally not indicated (eg, viral syndromes, influenza, uncomplicated zoster, and noninfectious gastroenteritis). For encounters with multiple diagnoses, Tier 1 took priority over Tier 2 and Tier 3. Overuse was defined as antibiotic administration for Tier 3 or no plausible indication; underuse was defined as no ED antibiotic administration for Tier 1. Tier 2 encounters were not classified as overuse or underuse. This operationalization measures the indication decision only and does not assess agent selection, dose, route, or duration.

### Outcomes

We evaluated LOS and clinical outcomes within 30 days of the index ED encounter: all-cause ED revisit, all-cause mortality, and CDI. LOS was measured from ED arrival to the final recorded discharge and therefore included the inpatient stay for admitted encounters; because of right-skewness, the primary model used log(1 + LOS). ED revisit was assessed only among encounters discharged from the ED, was all-cause, and was not classified according to whether the revisit diagnosis matched the index diagnosis. CDI was ascertained from diagnosis codes recorded within 30 days. We also evaluated a 5-level desirability of outcome ranking (DOOR) outcome (1 = alive with LOS at or below the median and no 30-day events, 2 = alive with LOS above the median and no events, 3 = 30-day ED revisit, 4 = CDI, 5 = death), consistent with DOOR principles [21]. Because hard adverse events were infrequent, LOS distinguished otherwise event-free encounters. In a sensitivity analysis, categories 1 and 2 were collapsed to create a 4-level DOOR outcome independent of the cohort median LOS.

### Covariates

Adjusted multivariable models accounted for patient-level covariates, including age, sex, race/ethnicity, marital status, insurance category, Charlson Comorbidity Index [22], triage systemic inflammatory response syndrome score, systolic blood pressure, heart rate [23], and travel time to the facility [19], and facility-level covariates, including staffed beds [16], teaching status [17], and rural–urban commuting area (RUCA)-based metropolitan/rural location [18]. Insurance records were mapped into 5 available categories; the final analytic insurance variable did not contain a distinct uninsured/self-pay category, so uninsured patients could not be analyzed separately.

### Statistical *Analysis*

We summarized baseline patient, facility, stewardship, contextual characteristics, and outcomes using medians (interquartile range) for continuous variables and counts (percentages) for categorical variables. To estimate adjusted associations of SDI and stewardship measures with outcomes, we fitted multivariable regression models. For logged LOS and DOOR score, we used ordinary least squares; for 30-day ED revisit, mortality, and CDI, we used linear probability models to estimate absolute risk differences. Models included state and calendar-month fixed effects and patient-clustered robust standard errors [24]. Overuse and underuse indicators were entered simultaneously, with encounters meeting neither criterion as the reference group. Reviewer-requested sensitivity analyses included modified Poisson models for adjusted relative risks, a 4-level DOOR outcome, log(LOS) among encounters with positive LOS to facilitate interpretation in minutes, complete-case models, and period-stratified analyses for the pre-pandemic period, early pandemic, and 2022–2024.

To assess whether guideline concordance mediated the association between SDI and each outcome, we performed regression-based mediation analysis using the natural effects framework [25, 26]. We estimated the total effect (TE) of SDI on each outcome and decomposed it into a natural indirect effect (NIE) through guideline concordance and a natural direct effect (NDE) not through concordance. The mediator model estimated guideline concordance as a function of SDI and covariates, and the outcome model estimated each outcome as a function of SDI, concordance, and the same covariates. Because both models were specified on additive scales without an SDI-by-concordance interaction, the NIE was estimated as the product of the SDI-to-concordance and concordance-to-outcome coefficients, and the NDE as the SDI coefficient from the outcome model. The proportion mediated was calculated as NIE/TE × 100%. Approximate 95% confidence intervals for TE and NIE were obtained using the delta method with cluster-robust covariance estimates. For binary outcomes, mediation effects were interpreted on the absolute risk-difference scale.

Missing covariate values were imputed using chained equations implemented in MICE, with predictive mean matching for continuous variables and multinomial logistic regression for categorical variables [27]. Primary analyses used a single completed dataset generated from the imputation procedure; therefore, model standard errors did not incorporate between-imputation variability. Cohort entry required nonmissing residence and facility linkage fields. Missingness by covariate, analysis-specific sample sizes, and complete-case sensitivity estimates are reported in the Supplementary Materials. The study was approved by the Baylor College of Medicine Institutional Review Board (H-56277) with waiver of informed consent. Analyses were conducted using R (R Foundation for Statistical Computing, Vienna, Austria).

## RESULTS

### Study Cohort and Observed Outcomes

The analytic cohort included 1 365 957 encounters (median age 75 years [IQR, 70–82]; 55% female; 76% White, 7.7% Black, 16% other race/ethnicity) (Table 1). Guideline-concordant antibiotics were administered in 83% of encounters; 9.5% met criteria for antibiotic overuse, and 7.4% for antibiotic underuse. Overall, 30-day ED revisit occurred in 19% of encounters among the subset with this outcome available (132 232/710 951), 30-day mortality was 2.5% (34 771/1 365 957), and 30-day CDI was 0.2% (2415/1 365 750) (Table 1). In the composite DOOR outcome, 44% of encounters were categorized as DOOR 1, 44% as DOOR 2, 9.4% as DOOR 3, 0.2% as DOOR 4, and 2.5% as DOOR 5.

**Table 1.** Patient, Facility, Stewardship, Contextual Characteristics, and Outcomes^a^

Characteristic	N = 1 365 957
Age, y	75 [70, 82]
Sex	
Female	757 642 (55%)
Male	608 315 (45%)
Race/ethnicity	
White	1 043 977 (76%)
Black	105 111 (7.7%)
Other	216 869 (16%)
Insurance category	
Commercial insurance—variable copays	667 622 (49%)
Government and military—low copays	155 440 (11%)
Medicaid—low or no copays	174 424 (13%)
Medicare advantage (managed care)—higher copays	100 502 (7.4%)
Traditional Medicare—moderate copays	267 969 (20%)
Charlson Comorbidity Index	4.00 [3.00, 6.00]
SIRS score at triage	
0	165 865 (12%)
1	574 030 (42%)
2	450 460 (33%)
3	159 880 (12%)
4	15 722 (1.2%)
Hospital RUCA category	
Rural	184 025 (13%)
Urban	1 181 932 (87%)
Social Deprivation Index (*z*-score)	−0.02 [−0.80, 0.79]
Travel time to hospital, min	17 [7, 24]
Guideline-concordant antibiotics	1 135 661 (83%)
Antibiotic overuse	129 446 (9.5%)
Antibiotic underuse	100 850 (7.4%)
Length of stay, d	0.24 [0.12, 2.86]
ED revisit within 30 d^b^	132 232 (19%)
Death within 30 d	34 771 (2.5%)
CDI within 30 d^c^	2415 (0.2%)
DOOR category (1 best, 5 worst)	
1	599 945 (44%)
2	599 943 (44%)
3	129 081 (9.4%)
4	2217 (0.2%)
5	34 771 (2.5%)

Abbreviations: CCI, Charlson Comorbidity Index; CDI, *Clostridioides difficile* infection; DOOR, desirability of outcome ranking; ED, emergency department; IQR, interquartile range; RUCA, rural–urban commuting area; SDI, Social Deprivation Index; SIRS, systemic inflammatory response syndrome.

^a^Data are presented as median [IQR] or No. (%), unless otherwise indicated.

^b^ED revisit within 30 d was assessed among encounters discharged from the ED with available 30-d revisit capture (132 232/710 951).

^c^CDI within 30 d was assessed among encounters with available outcome ascertainment (2415/1 365 750).

### Adjusted Associations of SDI and Guideline Concordance With Outcomes

Each 1-SD increase in neighborhood deprivation was associated with a small increase in LOS (0.3% longer; 95% CI, 0.1% to 0.4%), a 0.009-point worse DOOR score (95% CI, 0.007 to 0.010), a 0.59-percentage-point increase in 30-day ED revisit (95% CI, 0.48 to 0.70), and a 0.04-percentage-point increase in 30-day mortality (95% CI, 0.01 to 0.07). There was no meaningful association with 30-day CDI (0.04 cases per 10 000 encounters; 95% CI, −0.75 to 0.84) (Table 2).

**Table 2.** Adjusted Association of SDI and Guideline Concordance With Clinical Outcomes

Exposure	Outcome (Estimate with 95% CI)				
	LOS, % Change	DOOR, Δ Score	ED Revisit (30 d), Δ pp	Mortality (30 d), Δ pp	CDI (30 d), Δ per 10 000
SDI (per 1 SD higher)	0.3 (0.1, 0.4)	0.009 (0.007, 0.010)	0.59 (0.48, 0.70)	0.04 (0.01, 0.07)	0.04 (−0.75, 0.84)
Guideline-concordant versus nonconcordant	−22.3 (−22.6, −22.0)	−0.122 (−0.126, −0.118)	−1.74 (−2.02, −1.47)	−1.14 (−1.22, −1.06)	−13.12 (−15.46, −10.78)

Adjusted models report estimates with 95% CI. LOS is presented as percent change derived from log-LOS models: 100 × (e^β − 1). ED revisit and mortality are risk differences in percentage points (pp). CDI is risk difference per 10 000 encounters. DOOR is score difference.

Abbreviations: CDI = *Clostridioides difficile* infection; CI, confidence interval; DOOR, desirability of outcome ranking; LOS, length of stay; pp, percentage points; SDI, Social Deprivation Index.

Guideline-concordant antibiotic management was associated with a 22.3% shorter LOS (95% CI, 22.0% to 22.6% shorter), a 0.122-point better DOOR score (95% CI, 0.118 to 0.126 lower), a 1.74-percentage-point lower risk of 30-day ED revisit (95% CI, 1.47 to 2.02 lower), a 1.14-percentage-point lower risk of 30-day mortality (95% CI, 1.06 to 1.22 lower), and 13.12 fewer CDI cases per 10 000 encounters (95% CI, 10.78 to 15.46 fewer) (Table 2).

### Adjusted Associations of Antibiotic Overuse and Underuse With Outcomes

Relative to encounters meeting neither overuse nor underuse criteria, antibiotic overuse was associated with longer LOS (approximately 77% higher), a 0.262-point worse DOOR score, and higher absolute risks of 30-day ED revisit (1.02 percentage points), mortality (2.10 percentage points), and CDI (12.92 cases per 10 000 encounters) (Table 3).

**Table 3.** Adjusted Associations of Antibiotic Overuse and Underuse With Clinical Outcomes

Exposure	Outcome (Estimate with 95% CI)				
	LOS, % Change	DOOR, Δ score	ED Revisit (30 d), Δ pp	Mortality (30 d), Δ pp	CDI (30 d), Δ per 10 000
Antibiotic overuse	77.2 (76.3, 78.1)	0.262 (0.257, 0.267)	1.02 (0.47, 1.58)	2.10 (1.99, 2.21)	12.92 (9.93, 15.91)
Antibiotic underuse	−14.5 (−14.9, −14.1)	−0.057 (−0.063, −0.052)	2.03 (1.68, 2.38)	−0.09 (−0.16, −0.02)	13.38 (9.88, 16.87)

Estimates compare encounters with overuse or underuse with the reference group (encounters meeting neither criterion). Adjusted models report estimates with 95% CI. LOS is presented as percent change derived from log-LOS models: 100 × (e^β − 1). ED revisit and mortality are risk differences in percentage points (pp). CDI is expressed as cases per 10 000 encounters. DOOR is score difference.

Abbreviations: CDI, *Clostridioides difficile* infection; CI, confidence interval; DOOR, desirability of outcome ranking; LOS, length of stay; pp, percentage points.

Relative to encounters meeting neither overuse nor underuse criteria, antibiotic underuse was associated with shorter LOS (approximately 14% lower) and a 0.057-point better DOOR score, but higher absolute risks of 30-day ED revisit (2.03 percentage points) and CDI (13.38 cases per 10 000 encounters). Underuse was associated with a small reduction in 30-day mortality risk (0.09 percentage points), a pattern interpreted cautiously because of residual confounding, diagnosis heterogeneity, and possible delayed treatment after culture results (Table 3).

### Mediation of SDI Effects Through Guideline Concordance

In mediation analyses, guideline concordance accounted for only a modest fraction of the association between SDI and outcomes (Table 4). The proportion mediated (NIE/TE) reflects the fraction of the SDI–outcome association attributable to the pathway through guideline concordance. Estimated proportions mediated were 10.9% for LOS and 2.7% for DOOR, with smaller proportions for ED revisit (1.1%) and death (5.2%). For CDI, the indirect effect was statistically significant, but the TE estimate was near null; therefore, the mediated proportion should be interpreted cautiously.

**Table 4.** Mediation of SDI Effect on Outcomes Through Guideline Concordance

Outcome	Total Effect (TE)	Indirect Effect (NIE)	Proportion Mediated (%)	*P* Value (NIE)
LOS, % change	0.45 (0.29, 0.61)	0.05 (0.03, 0.07)	10.9	<.001
CDI (30 d), Δ per 10 000	0.175 (−0.618, 0.968)	0.025 (0.014, 0.035)	14.2	<.001
ED revisit (30 d), Δ percentage points	0.566 (0.458, 0.674)	0.006 (0.005, 0.008)	1.1	<.001
Death (30 d), Δ percentage points	0.043 (0.014, 0.071)	0.002 (0.001, 0.003)	5.2	<.001
DOOR (1–5), Δ score	0.0088 (0.0073, 0.0104)	0.0002 (0.0001, 0.0003)	2.7	<.001

TE = total effect of SDI (per 1 SD higher). NIE = natural indirect effect through guideline-concordant antibiotic management. Proportion mediated = NIE/TE × 100%.

NIE can be interpreted as the portion of the overall SDI–outcome association attributable to SDI-related differences in guideline concordance; the remainder (TE − NIE) reflects other pathways.

Scaling: LOS is expressed as a percent change; ED revisit, and death are absolute risk differences (percentage points); CDI is expressed as cases per 10 000 encounters; DOOR is the score difference.

Abbreviations: CDI, *Clostridioides difficile* infection; CI, confidence interval; DOOR, desirability of outcome ranking; LOS, length of stay; LPM, linear probability model; pp, percentage points; SD, standard deviation; SDI, Social Deprivation Index.

### Patterns Across SDI Quintiles

Across SDI quintiles (approximately 272 000–275 000 encounters per quintile), patient mix differed: compared with the lowest SDI quintile (Q1), the highest quintile (Q5) included a higher proportion of female patients (58% vs 53%), a higher proportion of Black patients (18% vs 2.4%), a lower proportion married (35% vs 60%), and a higher proportion insured by Medicaid (22% vs 5.8%) (Supplementary Table 1). Median LOS was similar across SDI quintiles (0.23 days in Q1 vs 0.25 days in Q5), while 30-day ED revisit increased from 17% (Q1) to 21% (Q5). DOOR distributions shifted modestly, with DOOR category 1 decreasing from 45% (Q1) to 42% (Q5) and DOOR category 3 increasing from 8.6% (Q1) to 10% (Q5). In contrast, stewardship patterns were stable across SDI quintiles: guideline-concordant antibiotics were ∼83% in every quintile, and overuse and underuse remained in narrow ranges (overuse 9.3%–9.7%, underuse 7.2%–7.7%) (Supplementary Table 2). Common diagnosis categories contributing to Tier 1 and Tier 3 classifications are summarized in Supplementary Table 3.

### Sensitivity Analyses

Modified Poisson models yielded adjusted relative risks for each 1−SD higher SDI of 1.033 (95% CI, 1.027–1.039) for ED revisit, 1.016 (95% CI, 1.004–1.027) for mortality, and 1.008 (95% CI, 0.964–1.054) for CDI. Corresponding adjusted relative risks for guideline concordance were 0.913 (95% CI, 0.900–0.926), 0.690 (95% CI, 0.674–0.707), and 0.559 (95% CI, 0.512–0.610), respectively (Supplementary Table 4).

Collapsing the 2 event-free DOOR categories produced similar associations and a mediated proportion of 0.8% (Supplementary Table 5). In an exact-log LOS sensitivity analysis that excluded 1064 encounters with zero recorded LOS (0.08%), each 1−SD higher SDI corresponded to approximately 2.2 additional minutes at the median LOS, whereas guideline concordance corresponded to approximately 132 fewer minutes (Supplementary Table 6). Period-stratified analyses showed generally similar directions of association, although magnitudes varied, particularly during the early COVID-19 pandemic (Supplementary Table 7).

Complete-case analyses retained 415 294 encounters for most outcomes and 174 671 for ED revisit. Associations between guideline concordance and outcomes remained directionally consistent; SDI associations with ED revisit and DOOR were also consistent, whereas the SDI–LOS association changed direction and the SDI–mortality association attenuated to the null (Supplementary Tables 8–9).

## DISCUSSION

In this large multicenter EHR-based cohort of 1 365 957 ED encounters among older adults, higher neighborhood deprivation showed statistically detectable but small absolute associations with several 30-day outcomes. Indication-level guideline concordance—particularly avoidance of overuse—was associated with larger differences in LOS, revisit, mortality, CDI, and DOOR, while mediating only a small fraction of deprivation-associated differences.

These findings extend our prior work in this ED network showing that higher area deprivation was associated with lower guideline-concordant prescribing, primarily through underuse [15]. Here, we show that concordance matters for downstream outcomes, but also that concordance alone explains only a limited share of deprivation-associated differences. National studies have shown that inappropriate antibiotic prescribing remains common in EDs and other ambulatory settings [5, 28]. Taken together, the current results suggest that stewardship is necessary for better outcomes but insufficient on its own to eliminate structurally patterned outcome gaps.

The association between SDI and worse outcomes is consistent with literature linking area-level disadvantage to barriers in access, continuity, timely evaluation, and follow-up [9–11, 29]. However, the adjusted SDI effects were small on the absolute scale. The exact-log sensitivity translated the LOS association to approximately 2 additional minutes at the cohort median, and some SDI estimates were sensitive to complete-case restriction. Thus, the SDI findings are most appropriately interpreted as modest population-level associations rather than large individual-level clinical effects.

Overuse demonstrated a consistent harmful association profile across outcomes, consistent with evidence that unnecessary antibiotic exposure contributes to adverse drug events, CDI, and AMR [1–4, 6, 7]. In contrast, underuse was associated with higher ED revisit and CDI risks but apparently shorter LOS and slightly lower mortality. We do not interpret these associations causally. They likely reflect residual confounding by indication, heterogeneity within Tier 1 diagnoses, coding-based misclassification, and differential disposition. In addition, because exposure was limited to antibiotics administered during the index ED encounter, some patients—particularly those evaluated for urinary infection—may have appropriately started therapy after culture or susceptibility results and therefore been classified as underuse. These findings support cautious, syndrome-specific validation of underuse metrics [20].

Mediation analyses suggest that guideline concordance explained only a small proportion of the total SDI–outcome association. This implies that deprivation-related differences are driven largely by pathways beyond the index antibiotic decision, including baseline health, outpatient access, diagnostic capacity, and structural constraints. The 4-level DOOR sensitivity analysis, which removed the median-LOS split between event-free encounters, yielded the same direction of association and similarly limited mediation. As with any causal mediation analysis, estimates rely on strong assumptions about unmeasured mediator–outcome confounding and should be considered hypothesis-generating [25].

These results have practical implications for ED stewardship programs seeking to integrate equity into routine quality improvement. Concordance metrics should be paired with explicit monitoring of overuse and underuse rather than overall antibiotic volume alone. Incorporating neighborhood context such as SDI into stewardship dashboards could help identify facilities or communities where high-deprivation patients experience higher revisit rates, longer LOS, or persistent overuse despite similar average concordance. In those settings, stewardship may be most effective when combined with diagnostic pathways for high-risk syndromes in older adults, improved discharge instructions, facilitated follow-up, and other supports that address barriers beyond the prescribing decision itself.

This study has limitations. Participating EDs differed in standards of care, diagnostic practices, and stewardship infrastructure, and the cohort was concentrated in 15 states with 76% of encounters among White patients; generalizability to regions and populations with different racial/ethnic composition or care systems may be limited. Exposures and outcomes were derived from routine EHR data, and residual confounding is likely, particularly for underuse. The diagnosis-tier framework captures indication-level concordance only and cannot adjudicate agent choice, dose, route, duration, evolving clinical information, or every appropriate exception. Discharge prescriptions and delayed antibiotic starts after culture results were not captured, potentially misclassifying some Tier 1 encounters as underuse. Professional guidance also evolved during the 10-year period, and clinicians may have followed newer syndrome-specific guidance not represented in the framework. ED revisits were all-cause, could not be classified as related or unrelated to the index diagnosis, and may have been missed outside participating facilities; CDI and other adverse events may also have been incompletely captured outside the network. The analytic insurance variable did not identify uninsured/self-pay patients separately. Missingness was substantial for several covariates; primary models used one MICE-generated completed dataset, so standard errors did not incorporate between-imputation variability, and some SDI estimates differed in complete-case analyses. Pandemic-period analyses showed variation in effect magnitude. Finally, mediation estimates remain sensitive to modeling assumptions and measurement error [25].

## CONCLUSIONS

Neighborhood deprivation showed small absolute associations with 30-day outcomes after ED encounters among older adults. Indication-level guideline concordance—especially avoidance of overuse—was associated with larger outcome differences but explained only a small share of deprivation-associated variation. Stewardship interventions should therefore be paired with broader diagnostic, follow-up, and equity-oriented system supports.

## Supplementary Material

ofag455_Supplementary_Data

## Supplementary Data

Supplementary materials are available at *Open Forum Infectious Diseases* online. Consisting of data provided by the authors to benefit the reader, the posted materials are not copyedited and are the sole responsibility of the authors, so questions or comments should be addressed to the corresponding author.

## References

[1] Murray CJ, Ikuta KS, Sharara F, et al Global burden of bacterial antimicrobial resistance in 2019: a systematic analysis. Lancet 2022; 399:629–55. 35065702 10.1016/S0140-6736(21)02724-0 PMC8841637

[2] Aguilar GR, Swetschinski LR, Weaver ND, et al The burden of antimicrobial resistance in the Americas in 2019: a cross-country systematic analysis. Lancet Reg Health Am 2023; 25:100551. 10.1016/j.lana.2023.100561 PMC10505822 37727594

[3] Jump RLP . *Clostridioides difficile* infection in older adults. Aging Health 2013; 9:403–14. 24955106 10.2217/ahe.13.37 PMC4061705

[4] Shehab N, Lovegrove MC, Geller AI, Rose KO, Weidle NJ, Budnitz DS. US emergency department visits for outpatient adverse drug events, 2013-2014. JAMA 2016; 316:2115–25. 27893129 10.1001/jama.2016.16201 PMC6490178

[5] Ladines-Lim JB, Fischer MA, Linder JA, Chua KP. Appropriateness of antibiotic prescribing in US emergency department visits, 2016-2021. Antimicrob Steward Healthc Epidemiol 2024; 4:e79. 38751940 10.1017/ash.2024.79 PMC11094377

[6] Hersh AL, King LM, Shapiro DJ, Hicks LA, Fleming-Dutra KE. Unnecessary antibiotic prescribing in US ambulatory care settings, 2010-2015. Clin Infect Dis 2021; 72:133–7. 32484505 10.1093/cid/ciaa667 PMC9377284

[7] Linder JA . Breaking the ambulatory antibiotic prescribing cycle with all-antibiotic stewardship, patient stewardship, and visit stewardship. Clin Infect Dis 2021; 73:e1680–3. 32776131 10.1093/cid/ciaa1170 PMC8492153

[8] Robert Graham Center . Social Deprivation Index (SDI). Available at: https://www.graham-center.org/maps-data-tools/social-deprivation-index.html. Accessed 16 September 2025.

[9] Butler DC, Petterson S, Phillips RL, Bazemore AW. Measures of social deprivation that predict health care access and need within a rational area of primary care service delivery. Health Serv Res 2013; 48:539–59. 22816561 10.1111/j.1475-6773.2012.01449.x PMC3626349

[10] Mullachery PH, Li R, Melly S, et al Inequities in spatial accessibility to COVID-19 testing in 30 large US cities. Soc Sci Med 2022; 310:115307. 36049353 10.1016/j.socscimed.2022.115307 PMC9420026

[11] Dhand A, Reeves MJ, Mu Y, et al Mapping the ecological terrain of stroke prehospital delay: a nationwide registry study. Stroke 2024; 55:1507–16. 38787926 10.1161/STROKEAHA.123.045521 PMC11299104

[12] Al Mohajer M, Slusky D, Nix D, Nicodemo C. Investigating socioeconomic deprivation and antibiotic prescribing among older Medicare patients using an instrumental variable approach. Antimicrob Steward Healthc Epidemiol 2025; 5:e110. 40395946 10.1017/ash.2025.185 PMC12089662

[13] Seibert AM, Hersh AL, Patel PK, et al Urgent-care antibiotic prescribing: an exploratory analysis to evaluate health inequities. Antimicrob Steward Healthc Epidemiol 2022; 2:e184. 36406162 10.1017/ash.2022.329 PMC9672912

[14] Young EH, Strey KA, Lee GC, et al National disparities in antibiotic prescribing by race, ethnicity, age group, and sex in United States ambulatory care visits, 2009 to 2016. Antibiotics (Basel) 2022; 12:51. 36671252 10.3390/antibiotics12010051 PMC9854843

[15] Al Mohajer M, Slusky D, Nix D, Allel K, Shay S, Nicodemo C. Antibiotic guideline concordance and area deprivation in the US emergency departments, 2015-2024. Infect Control Hosp Epidemiol 2026; doi:10.1017/ice.2026.10410 41717790

[16] American Hospital Association . AHA annual survey database. Chicago, IL: American Hospital Association, 2025.

[17] Centers for Medicare & Medicaid Services . Open payments: teaching hospitals. Available at: https://openpaymentsdata.cms.gov/dataset/Teaching-Hospitals/rrge-4×3b/about_data. Accessed 16 September 2025.

[18] USDA Economic Research Service . Rural-urban commuting area codes. Available at: https://www.ers.usda.gov/data-products/rural-urban-commuting-area-codes.aspx. Accessed 16 September 2025.

[19] Giraud T . osrm: interface between R and the OpenStreetMap-based routing service OSRM. R package version 4.0.2. **2024**. Available at: https://cran.r-project.org/web/packages/osrm. Accessed 16 September 2025.

[20] Chua KP, Fischer MA, Linder JA. Appropriateness of outpatient antibiotic prescribing among privately insured US patients: ICD-10-CM based cross-sectional study. Br Med J 2019; 364:k5092. 30651273 10.1136/bmj.k5092 PMC6334180

[21] Evans SR, Rubin D, Follmann D, et al Desirability of outcome ranking (DOOR) and response adjusted for duration of antibiotic risk (RADAR). Clin Infect Dis 2015; 61:800–6. 26113652 10.1093/cid/civ495 PMC4542892

[22] Charlson ME, Pompei P, Ales KL, MacKenzie CR. A new method of classifying prognostic comorbidity in longitudinal studies: development and validation. J Chronic Dis 1987; 40:373–83. 3558716 10.1016/0021-9681(87)90171-8

[23] Bone RC, Balk RA, Cerra FB, et al Definitions for sepsis and organ failure and guidelines for the use of innovative therapies in sepsis. Chest 1992; 101:1644–55. 1303622 10.1378/chest.101.6.1644

[24] Cameron AC, Miller DL. A practitioner's guide to cluster-robust inference. J Hum Resour 2015; 50:317–72.

[25] Imai K, Keele L, Tingley D. A general approach to causal mediation analysis. Psychol Methods 2010; 15:309–34. 20954780 10.1037/a0020761

[26] Tingley D, Yamamoto T, Hirose K, Keele L, Imai K. Mediation: R package for causal mediation analysis. J Stat Softw 2014; 59:1–38.26917999

[27] Van Buuren S, Groothuis-Oudshoorn K. Mice: multivariate imputation by chained equations in R. J Stat Softw 2011; 45:1–67.

[28] Palms DL, Hicks LA, Bartoces M, et al Comparison of antibiotic prescribing in retail clinics, urgent care centers, emergency departments, and traditional ambulatory care settings in the United States. JAMA Intern Med 2018; 178:1267–9. 30014128 10.1001/jamainternmed.2018.1632 PMC6142958

[29] Morenz AM, Liao JM, Au DH, Hayes SA. Area-level socioeconomic disadvantage and health care spending: a systematic review. JAMA Netw Open 2024; 7:e2356121. 38358740 10.1001/jamanetworkopen.2023.56121 PMC10870184

