# Supplementary material for: Neighborhood Deprivation, Antibiotic Prescribing Concordance, and 30-Day Outcomes After Emergency Department Encounters in Older Adults
Source: Open Forum Infect Dis. 2026 Jul 23;13(7):ofag455. doi: 10.1093/ofid/ofag455 (PMC13416734; doi:10.1093/ofid/ofag455)
Supplement: ofag455_Supplementary_Data [file ofag455_supplementary_data.docx]

Supplementary Material

Supplementary Methods and Notes

Antibiotic indication tiers were assigned using the encounter-level ICD-9/ICD-10 framework from our related ED-network study. Antibiotic exposure represented antibacterial administrations recorded during the index ED encounter; discharge prescriptions and delayed post-discharge starts were not captured. The framework was informed principally by Infectious Diseases Society of America guidance available before December 2024 and assessed the indication decision rather than agent selection, dose, route, or duration. Tier 1 represented diagnoses for which systemic antibiotics are generally indicated, Tier 2 diagnoses requiring clinical context, and Tier 3 diagnoses for which antibiotics are generally not indicated. For encounters with multiple diagnoses, Tier 1 took priority over Tier 2 and Tier 3.

**Supplementary Table 1.** Selected patient, facility, stewardship, contextual characteristics, and outcomes by SDI quintile

**Supplementary Table 2.** Stewardship patterns by SDI quintile

Missing-data note: Residence and facility linkage fields were required for cohort entry and were not imputed. Other missing covariates were filled using chained equations implemented in MICE. Primary analyses used one completed dataset generated from the imputation procedure; model standard errors therefore did not incorporate between-imputation variability. Complete-case analyses are reported as sensitivity analyses.

**Supplementary Table 1.** Selected patient, facility, stewardship, contextual characteristics, and outcomes by SDI quintile

| **Characteristic** | **SDI quintile** |  |  |  |  |
| --- | --- | --- | --- | --- | --- |
|  | **Q1** N = 273,229*^1^* | **Q2** N = 274,740*^1^* | **Q3** N = 271,914*^1^* | **Q4** N = 273,150*^1^* | **Q5** N = 272,924*^1^* |
| **Age, years** | 76 [71, 82] | 76 [70, 82] | 76 [70, 82] | 75 [70, 82] | 74 [69, 81] |
| **Sex** |  |  |  |  |  |
| Female | 144,359 (53%) | 149,853 (55%) | 150,873 (55%) | 154,860 (57%) | 157,697 (58%) |
| Male | 128,870 (47%) | 124,887 (45%) | 121,041 (45%) | 118,290 (43%) | 115,227 (42%) |
| **Race/ethnicity** |  |  |  |  |  |
| White | 225,641 (83%) | 225,284 (82%) | 216,533 (80%) | 207,449 (76%) | 169,070 (62%) |
| Black | 6,490 (2.4%) | 10,664 (3.9%) | 14,396 (5.3%) | 23,109 (8.5%) | 50,452 (18%) |
| Other | 41,098 (15%) | 38,792 (14%) | 40,985 (15%) | 42,592 (16%) | 53,402 (20%) |
| **Insurance category** |  |  |  |  |  |
| Commercial Insurance - Variable Copays | 140,890 (52%) | 137,300 (50%) | 130,706 (48%) | 129,823 (48%) | 128,903 (47%) |
| Government & Military - Low Copays | 32,524 (12%) | 32,983 (12%) | 31,059 (11%) | 32,485 (12%) | 26,389 (9.7%) |
| Medicaid - Low or No Copays | 15,923 (5.8%) | 24,256 (8.8%) | 32,134 (12%) | 42,175 (15%) | 59,936 (22%) |
| Medicare Advantage (Managed Care) - Higher Copays | 20,074 (7.3%) | 20,507 (7.5%) | 21,017 (7.7%) | 20,207 (7.4%) | 18,697 (6.9%) |
| Traditional Medicare - Moderate Copays | 63,818 (23%) | 59,694 (22%) | 56,998 (21%) | 48,460 (18%) | 38,999 (14%) |
| **Charlson Comorbidity Index** | 4.00 [3.00, 5.00] | 4.00 [3.00, 5.00] | 4.00 [3.00, 6.00] | 4.00 [3.00, 6.00] | 4.00 [3.00, 6.00] |
| **SIRS score at triage** |  |  |  |  |  |
| 0 | 32,848 (12%) | 32,843 (12%) | 32,458 (12%) | 33,407 (12%) | 34,309 (13%) |
| 1 | 116,353 (43%) | 115,693 (42%) | 113,993 (42%) | 114,509 (42%) | 113,482 (42%) |
| 2 | 88,661 (32%) | 90,515 (33%) | 90,230 (33%) | 90,650 (33%) | 90,404 (33%) |
| 3 | 32,094 (12%) | 32,447 (12%) | 32,076 (12%) | 31,594 (12%) | 31,669 (12%) |
| 4 | 3,273 (1.2%) | 3,242 (1.2%) | 3,157 (1.2%) | 2,990 (1.1%) | 3,060 (1.1%) |
| **Hospital RUCA category** |  |  |  |  |  |
| Rural | 16,257 (5.9%) | 34,884 (13%) | 41,159 (15%) | 54,716 (20%) | 37,009 (14%) |
| **Social Deprivation Index (standardized analytic score)** | −1.21 [−1.47, −1.04] | −0.59 [−0.73, −0.44] | −0.03 [−0.16, 0.08] | 0.53 [0.39, 0.70] | 1.24 [1.05, 1.47] |
| **Travel time to hospital, minutes** | 18 [9, 25] | 18 [8, 25] | 18 [7, 25] | 16 [4, 24] | 14 [3, 21] |
| **Guideline-concordant antibiotics** | 227,052 (83%) | 228,433 (83%) | 226,342 (83%) | 226,922 (83%) | 226,912 (83%) |
| **Antibiotic overuse** | 26,438 (9.7%) | 26,253 (9.6%) | 25,338 (9.3%) | 25,326 (9.3%) | 26,091 (9.6%) |
| **Antibiotic underuse** | 19,739 (7.2%) | 20,054 (7.3%) | 20,234 (7.4%) | 20,902 (7.7%) | 19,921 (7.3%) |
| **Length of stay, days** | 0.23 [0.12, 2.78] | 0.23 [0.12, 2.80] | 0.24 [0.12, 2.87] | 0.23 [0.12, 2.79] | 0.25 [0.13, 3.02] |
| **ED revisit within 30 days***^2^* | 24,148 (17%) | 25,591 (18%) | 26,257 (18%) | 27,592 (19%) | 28,644 (21%) |
| **Death within 30 days** | 6,718 (2.5%) | 6,942 (2.5%) | 7,176 (2.6%) | 6,958 (2.5%) | 6,977 (2.6%) |
| **CDI within 30 days** | 467 (0.2%) | 481 (0.2%) | 519 (0.2%) | 478 (0.2%) | 470 (0.2%) |
| **DOOR category (1 best, 5 worst)** |  |  |  |  |  |
| 1 | 121,830 (45%) | 122,828 (45%) | 119,526 (44%) | 121,565 (45%) | 114,196 (42%) |
| 2 | 120,710 (44%) | 119,579 (44%) | 119,180 (44%) | 117,200 (43%) | 123,274 (45%) |
| 3 | 23,540 (8.6%) | 24,947 (9.1%) | 25,566 (9.4%) | 26,986 (9.9%) | 28,042 (10%) |
| 4 | 431 (0.2%) | 444 (0.2%) | 466 (0.2%) | 441 (0.2%) | 435 (0.2%) |
| 5 | 6,718 (2.5%) | 6,942 (2.5%) | 7,176 (2.6%) | 6,958 (2.5%) | 6,977 (2.6%) |

^1 Data are presented as median [IQR] or No. (%), unless otherwise indicated. SDI quintiles are ordered from Q1 (lowest deprivation) to Q5 (highest deprivation). The source SDI ranges from 1 to 100; the analytic score was standardized, so negative values indicate deprivation below the cohort mean and positive values indicate deprivation above the cohort mean. Median observed source scores across Q1–Q5 were 12, 31, 50, 69, and 89, respectively.

^2 ED revisit within 30 days was assessed among encounters discharged from the ED with available 30-day revisit capture (denominator varies by quintile).

*Abbreviations:* CCI = Charlson Comorbidity Index; DOOR = desirability of outcome ranking; ED = emergency department; F = female; IQR = interquartile range; M = male; Q = quintile; RUCA = Rural–Urban Commuting Area; SDI = Social Deprivation Index; SIRS = systemic inflammatory response syndrome; CDI = Clostridioides difficile infection.

| **Supplementary Table 2. Stewardship patterns by SDI quintile** | | | | |
| --- | --- | --- | --- | --- |
| SDI_quintile | N | Guideline-concordant antibiotics, % | Antibiotic overuse, % | Antibiotic underuse, % |
| Q1 | 273,229 | 83.1 | 9.7 | 7.2 |
| Q2 | 274,740 | 83.1 | 9.6 | 7.3 |
| Q3 | 271,914 | 83.2 | 9.3 | 7.4 |
| Q4 | 273,150 | 83.1 | 9.3 | 7.7 |
| Q5 | 272,924 | 83.1 | 9.6 | 7.3 |
| Percentages are among all encounters; overuse/underuse are coded 0 when unmeasured. | | | | |
| *Abbreviations:* SDI = Social Deprivation Index. | | | | |

**Supplementary Sensitivity Analysis Methods**

We conducted five reviewer-requested sensitivity analyses. First, modified Poisson fixed-effects models estimated adjusted relative risks for binary outcomes while retaining the same covariates, state and calendar-month fixed effects, and patient-clustered standard errors as the primary models. Second, DOOR categories 1 and 2 were collapsed to create a 4-level outcome independent of the cohort median LOS. Third, LOS was modeled as log(LOS) among encounters with positive recorded LOS; effects were translated to approximate minutes at the cohort median LOS. Fourth, models were stratified into pre-pandemic (before March 1, 2020), early-pandemic (March 1, 2020–December 31, 2021), and later (2022–2024) periods. Fifth, complete-case analyses excluded observations flagged as imputed for any modeled covariate.

**Supplementary Table 3. Common diagnosis categories contributing to Tier 1 and Tier 3 classifications**

| **Tier** | **Common diagnosis category** | **Representative ICD-9/ICD-10 codes** |
| --- | --- | --- |
| Tier 1: antibiotics generally indicated | Urinary tract infection and acute cystitis | N39.0, N30.00, N30.01, 599.0 |
| Tier 1: antibiotics generally indicated | Bacterial or unspecified pneumonia | J18.9, J18.0, J18.1 |
| Tier 1: antibiotics generally indicated | Sepsis | A41.9 and related sepsis codes |
| Tier 1: antibiotics generally indicated | Cellulitis and other bacterial skin/soft-tissue infection | L03.11x, L03.21x and related codes |
| Tier 3: antibiotics generally not indicated | COVID-19 and viral respiratory syndromes | U07.1, J12.82, B34.9 |
| Tier 3: antibiotics generally not indicated | Influenza | J10.1 |
| Tier 3: antibiotics generally not indicated | Uncomplicated herpes zoster | B02.9 |
| Tier 3: antibiotics generally not indicated | Noninfectious gastroenteritis/colitis or other noninfectious presentations | K52.9 and noninfectious diagnosis codes |

Note: Categories summarize the most frequent diagnosis-code groups in the audit and are illustrative rather than an exhaustive code list. The encounter-level tier was assigned using the highest-priority applicable diagnosis.

**Supplementary Table 4. Adjusted relative risks from modified Poisson sensitivity models**

| **Outcome** | **Exposure** | **Adjusted RR (95% CI)** | **Model N** |
| --- | --- | --- | --- |
| 30-day ED revisit | SDI, per 1 SD higher | 1.033 (1.027, 1.039) | 710,951 |
| 30-day ED revisit | Guideline-concordant vs non-concordant | 0.913 (0.900, 0.926) | 710,951 |
| 30-day mortality | SDI, per 1 SD higher | 1.016 (1.004, 1.027) | 1,365,957 |
| 30-day mortality | Guideline-concordant vs non-concordant | 0.690 (0.674, 0.707) | 1,365,957 |
| 30-day CDI | SDI, per 1 SD higher | 1.008 (0.964, 1.054) | 1,328,415 |
| 30-day CDI | Guideline-concordant vs non-concordant | 0.559 (0.512, 0.610) | 1,328,415 |

Note: Models used the same covariates, state and calendar-month fixed effects, and patient-clustered standard errors as the primary analyses. ED revisit was restricted to ED discharges.

**Supplementary Table 5. Four-level DOOR sensitivity analysis**

| **Analysis** | **Exposure/effect** | **Estimate (95% CI) or mediation result** | **N** |
| --- | --- | --- | --- |
| Adjusted association | SDI, per 1 SD higher | 0.0050 (0.0040, 0.0060) | 1,365,957 |
| Adjusted association | Guideline-concordant vs non-concordant | -0.0251 (-0.0279, -0.0224) | 1,365,957 |
| Mediation | Indirect effect through concordance | NIE 0.000040; proportion mediated 0.8% | 1,365,957 |

Note: The 4-level DOOR outcome combined the two event-free categories, followed by ED revisit, CDI, and death.

**Supplementary Table 6. Sensitivity analysis using log(LOS) among encounters with positive LOS**

| **Exposure** | **% change in LOS (95% CI)** | **Approximate minutes at median LOS (95% CI)** | **N** |
| --- | --- | --- | --- |
| SDI, per 1 SD higher | 0.64% (0.30%, 0.98%) | 2.2 (1.0, 3.3) | 1,364,893 |
| Guideline-concordant vs non-concordant | -38.95% (-39.44%, -38.46%) | -132.4 (-134.1, -130.8) | 1,364,893 |

Note: The reference median LOS was 0.236 days (approximately 5.67 hours). This analysis excluded 1,064 encounters (0.08%) with a recorded LOS of zero.

**Supplementary Table 7. Period-stratified sensitivity analyses**

| **Period** | **Outcome** | **Exposure** | **Estimate (95% CI)** | **Unit** | **N** |
| --- | --- | --- | --- | --- | --- |
| Pre-pandemic | LOS | SDI, per 1 SD higher | 1.623 (0.852, 2.401) | % change | 351,771 |
| Pre-pandemic | LOS | Guideline-concordant vs non-concordant | -27.945 (-29.132, -26.738) | % change | 351,771 |
| Pre-pandemic | DOOR (1-5) | SDI, per 1 SD higher | 0.014 (0.010, 0.017) | score difference | 352,103 |
| Pre-pandemic | DOOR (1-5) | Guideline-concordant vs non-concordant | -0.096 (-0.103, -0.089) | score difference | 352,103 |
| Pre-pandemic | 30-day ED revisit | SDI, per 1 SD higher | 0.800 (0.600, 1.000) | percentage points | 212,937 |
| Pre-pandemic | 30-day ED revisit | Guideline-concordant vs non-concordant | -2.454 (-2.913, -1.995) | percentage points | 212,937 |
| Pre-pandemic | 30-day mortality | SDI, per 1 SD higher | 0.042 (-0.014, 0.098) | percentage points | 352,103 |
| Pre-pandemic | 30-day mortality | Guideline-concordant vs non-concordant | -0.735 (-0.882, -0.589) | percentage points | 352,103 |
| Pre-pandemic | 30-day CDI | SDI, per 1 SD higher | 0.730 (-1.533, 2.994) | per 10,000 | 352,056 |
| Pre-pandemic | 30-day CDI | Guideline-concordant vs non-concordant | -33.989 (-40.458, -27.519) | per 10,000 | 352,056 |
| Early pandemic | LOS | SDI, per 1 SD higher | 0.656 (-0.131, 1.448) | % change | 240,942 |
| Early pandemic | LOS | Guideline-concordant vs non-concordant | -49.933 (-50.897, -48.951) | % change | 240,942 |
| Early pandemic | DOOR (1-5) | SDI, per 1 SD higher | 0.009 (0.005, 0.013) | score difference | 241,141 |
| Early pandemic | DOOR (1-5) | Guideline-concordant vs non-concordant | -0.189 (-0.199, -0.179) | score difference | 241,141 |
| Early pandemic | 30-day ED revisit | SDI, per 1 SD higher | 0.500 (0.263, 0.736) | percentage points | 143,521 |
| Early pandemic | 30-day ED revisit | Guideline-concordant vs non-concordant | -1.531 (-2.178, -0.884) | percentage points | 143,521 |
| Early pandemic | 30-day mortality | SDI, per 1 SD higher | 0.080 (0.001, 0.158) | percentage points | 241,141 |
| Early pandemic | 30-day mortality | Guideline-concordant vs non-concordant | -2.716 (-2.956, -2.476) | percentage points | 241,141 |
| Early pandemic | 30-day CDI | SDI, per 1 SD higher | -1.355 (-2.998, 0.288) | per 10,000 | 241,078 |
| Early pandemic | 30-day CDI | Guideline-concordant vs non-concordant | -4.792 (-9.233, -0.352) | per 10,000 | 241,078 |
| 2022-2024 | LOS | SDI, per 1 SD higher | 0.274 (-0.119, 0.669) | % change | 772,180 |
| 2022-2024 | LOS | Guideline-concordant vs non-concordant | -38.304 (-38.906, -37.697) | % change | 772,180 |
| 2022-2024 | DOOR (1-5) | SDI, per 1 SD higher | 0.007 (0.005, 0.009) | score difference | 772,713 |
| 2022-2024 | DOOR (1-5) | Guideline-concordant vs non-concordant | -0.104 (-0.109, -0.100) | score difference | 772,713 |
| 2022-2024 | 30-day ED revisit | SDI, per 1 SD higher | 0.562 (0.412, 0.712) | percentage points | 354,493 |
| 2022-2024 | 30-day ED revisit | Guideline-concordant vs non-concordant | -1.289 (-1.692, -0.887) | percentage points | 354,493 |
| 2022-2024 | 30-day mortality | SDI, per 1 SD higher | 0.026 (-0.011, 0.062) | percentage points | 772,713 |
| 2022-2024 | 30-day mortality | Guideline-concordant vs non-concordant | -0.762 (-0.867, -0.657) | percentage points | 772,713 |
| 2022-2024 | 30-day CDI | SDI, per 1 SD higher | 0.150 (-0.665, 0.965) | per 10,000 | 772,616 |
| 2022-2024 | 30-day CDI | Guideline-concordant vs non-concordant | -4.997 (-7.333, -2.662) | per 10,000 | 772,616 |

Note: Periods were pre-pandemic (before March 1, 2020), early pandemic (March 1, 2020–December 31, 2021), and 2022–2024.

**Supplementary Table 8A. Covariate missingness before chained-equation imputation**

| **Variable** | **N missing/imputed** | **% of cohort** |
| --- | --- | --- |
| SDI | 507,805 | 37.2% |
| Insurance | 134,516 | 9.8% |
| Marital status | 16,837 | 1.2% |
| Race/ethnicity | 27,815 | 2.0% |
| Sex | 33 | 0.0% |
| Travel time | 91,944 | 6.7% |
| Systolic blood pressure | 531,831 | 38.9% |
| SIRS score | 534,829 | 39.2% |

**Supplementary Table 8B. Analysis-specific sample sizes**

| **Analysis** | **N** |
| --- | --- |
| LOS primary | 1,365,957 |
| CDI primary | 1,365,750 |
| ED revisit primary | 710,951 |
| Mortality primary | 1,365,957 |
| DOOR primary | 1,365,957 |
| LOS exact-log sensitivity | 1,364,893 |
| DOOR4 sensitivity | 1,365,957 |

**Supplementary Table 9. Complete-case sensitivity analyses**

| **Outcome** | **Exposure** | **Estimate (95% CI)** | **Unit** | **N** |
| --- | --- | --- | --- | --- |
| LOS | SDI, per 1 SD higher | -0.352 (-0.606, -0.098) | % change | 415,294 |
| LOS | Guideline-concordant vs non-concordant | -24.685 (-25.164, -24.202) | % change | 415,294 |
| DOOR (1-5) | SDI, per 1 SD higher | 0.005 (0.003, 0.008) | score difference | 415,294 |
| DOOR (1-5) | Guideline-concordant vs non-concordant | -0.114 (-0.120, -0.108) | score difference | 415,294 |
| 30-day ED revisit | SDI, per 1 SD higher | 0.748 (0.527, 0.969) | percentage points | 174,671 |
| 30-day ED revisit | Guideline-concordant vs non-concordant | -1.578 (-2.177, -0.978) | percentage points | 174,671 |
| 30-day mortality | SDI, per 1 SD higher | -0.017 (-0.071, 0.036) | percentage points | 415,294 |
| 30-day mortality | Guideline-concordant vs non-concordant | -1.193 (-1.348, -1.038) | percentage points | 415,294 |
| 30-day CDI | SDI, per 1 SD higher | -0.457 (-1.666, 0.753) | per 10,000 | 415,241 |
| 30-day CDI | Guideline-concordant vs non-concordant | -4.054 (-7.362, -0.745) | per 10,000 | 415,241 |

Note: Complete-case analyses excluded encounters flagged as imputed for any modeled covariate. These estimates should be interpreted in light of the substantial reduction in sample size.

Supplementary abbreviations: CDI, Clostridioides difficile infection; CI, confidence interval; DOOR, desirability of outcome ranking; ED, emergency department; LOS, length of stay; NIE, natural indirect effect; RR, relative risk; SDI, Social Deprivation Index.
